# A protocol for estimating health burden posed by early life exposure to ambient fine particulate matter and its heavy metal composition: a mother–child birth (ELitE) cohort from Central India

**DOI:** 10.3389/fpubh.2025.1485417

**Published:** 2025-05-20

**Authors:** Tanwi Trushna, Vikas Yadav, Uday Kumar Mandal, Vishal Diwan, Rajnarayan R. Tiwari, Rajesh Ahirwar, Dharma Raj, Sindhuprava Rana, Suchitra Vishwambhar Surve, Sagnik Dey, Yogesh Damodar Sabde

**Affiliations:** ^1^Department of Environmental Health and Epidemiology, ICMR-National Institute for Research in Environmental Health, Bhopal, India; ^2^Faculty of Medical Sciences, Academy of Scientific and Innovative Research (AcSIR), Ghaziabad, India; ^3^Department of Environmental Monitoring and Exposure Assessment (Water and Soil), National Institute for Research in Environmental Health, Bhopal, India; ^4^Department of Environmental Biochemistry, ICMR-National Institute for Research in Environmental Health, Bhopal, India; ^5^Department of Biostatistics and Bioinformatics, ICMR-National Institute for Research in Environmental Health, Bhopal, India; ^6^Department of Child Health Research, ICMR - National Institute for Research in Reproductive and Child Health, Mumbai, India; ^7^Centre for Atmospheric Sciences, Indian Institute of Technology (IIT-Delhi), Delhi, India; ^8^Centre of Excellence for Research on Clean Air, Indian Institute of Technology (IIT-Delhi), Delhi, India

**Keywords:** acute respiratory infection, air pollution, birth cohort, child development, growth, heavy metals, India, particulate matter

## Abstract

**Background:**

Pregnant women and children are vulnerable to air pollution-related adverse health effects, especially those residing in low-resource and high-exposure settings like India. However, evidence regarding the effects of early-life exposure to air particulate matter (PM) on childhood growth/developmental trajectory is contradictory; evidence about specific constituents of PM, like heavy metals, is limited. Similarly, there are few Indian cohorts investigating PM exposure and the incidence of acute respiratory infection during infancy. This study protocol aims to fill these critical gaps in knowledge.

**Methods:**

We aim to establish a mother–child birth cohort through the enrolment of 1,566 pregnant women residing in two urban areas of central India. Antenatally, we will collect socioeconomic, demographic, and clinical information and details of confounding variables from these pregnant women, who will then be followed up until childbirth to assess their exposure to air PM. Biomonitoring will also be conducted to evaluate heavy metal exposure. At birth, pregnancy outcomes will be noted, followed by postnatal follow-up of live-born children until the first year of life to assess their achievement of growth/development milestones and exposure to pollutants. We will also estimate the incidence of Acute Respiratory Infections (ARI) during infancy.

**Discussion:**

This manuscript describes the protocol for an Indian mother–child air pollution birth cohort study that aims to generate comprehensive evidence regarding the adverse effects of early-life (i.e., both pre- and post-natal) exposure to air PM and its constituent heavy metals among Indian children. This study will provide an epidemiological basis for further understanding in this context. Finally, by reporting our carefully planned study methods/outcome measures, which are comparable to those of published and ongoing birth cohorts, we aim to serve as the starting point for similar cohorts in the future, which, when considered together, would generate enough evidence to facilitate context-specific policy-making and development of appropriate prevention and mitigation strategies.

## 1 Introduction

Air pollution is the most significant worldwide threat to human health and life expectancy, with 7 million global deaths being attributable to its exposure (1, 2). The United Nations Environment Programme (UNEP) reported that the vast majority of people worldwide reside in places where the concentration of particulate matter (PM) pollutants in the air exceeds the stringent permissible limits prescribed in the 2021 air quality guidelines of the World Health Organization (WHO) (3, 4). People living in India and other low- and middle-income countries, particularly pregnant women and children, are at significantly higher health risk (5–7). Therefore, the WHO– United Nations Children’s Fund (UNICEF)–Lancet Commission, in its 2020 report focussing on the future of the global child population, has stressed evidence generation and subsequent interventions to safeguard the health of children, especially in high pollution exposure settings, to expedite the fulfillment of the 48 child-related Sustainable Development Goal (SDG) indicators (8, 9).

Air pollution adversely affects pregnancy, fetal growth, and development (10–12). However, evidence regarding the effects of early-life (i.e., pre- and post-natal) exposure to air particulate matter on the childhood growth trajectory is contradictory. Some studies demonstrate an increased relative risk of childhood stunting, wasting, and being underweight (13–15). At the same time, others have also reported a higher risk of childhood obesity and raised body mass index (BMI) (16, 17). Even among infants, published evidence is contradictory. For example, a Colorado-based prospective cohort study reported higher adiposity among exposed infants at the 5th-month follow-up (18). Similar results were reported by a Chinese birth cohort (19). In contrast, a cohort study from Ghana reported lower length-for-age (stunting) and weight-for-length (wasting) z-scores among exposed infants (15). On the other hand, a recent randomized trial conducted in four low- and middle-income countries that substituted biomass burning with clean cooking fuel, leading to a reduction in antenatal personal exposure levels, reported no difference in the risk of stunting in infants (20). Therefore, further research is required to provide confirmatory evidence. This ambiguity in evidence might be because few studies have investigated the differential effect of chemical constituents of particulate matter, which are a heterogeneous mixture of multiple components with varying toxicity profiles (21). Particularly, there is a paucity of longitudinal research investigating the association between early-life exposure to multiple heavy metals and aberrations in childhood growth or development (22, 23). Considering the magnitude of the public health burden posed by air pollution and child growth/developmental abnormalities in low- and middle-income countries such as India (24), where annually more than 250 million under-five children fail to attain their optimum developmental potential (25), this study protocol aims to generate comprehensive evidence in this context to complement the limited India-specific evidence published so far (26–28).

Another adverse effect of prenatal air pollution exposure is the alteration of immune mechanisms in children (29–31), which can increase the risk of infectious diseases such as acute respiratory infections (ARIs). Evidence supporting this link is mainly based on studies conducted in high-income countries (32). The burden of childhood ARI is enormous in India, which is one of the top 15 countries globally in terms of the prevalence of ARI and subsequent childhood mortality, with 0.4 million under-five children dying annually from ARI-related diseases (33). However, Indian evidence on air pollution-induced childhood ARI is mainly limited to cross-sectional surveys or ecological retrospective data analysis. Further, these studies have used proxy measures such as questionnaire data to elicit either household exposures (34, 35) or ambient air pollution (36, 37). Thus, considering these knowledge gaps, this study protocol also focuses on identifying the association of variation in the incidence of ARI till one year of age among children exposed during early life to different levels of air PM and its heavy metal content.

## 2 Aims and objectives

We aim to establish an urban mother–child birth cohort in central India. The data collected from this cohort will enable us to understand the health burden posed by early life exposure (i.e., pre- and post-natal) to air PM and its heavy metal composition in Indian children and the role of such environmental exposure in the multifactorial aetiology of our chosen study outcomes. The specific objectives being addressed in this research are:

1. To investigate the variation in achievement of growth/developmental milestones during the infancy period of children attributable to different levels of early life exposure to air pollution
2. To assess the incidence of acute respiratory infections during the infancy period of children attributable to different levels of early-life exposure to air pollution
3. To find the association of exposure to selected heavy metals (as represented by blood concentration) among pregnant women /children with assessed morbidity outcomes of children.

## 3 Materials and methods

During the drafting of this manuscript, details were reported as per the STROBE checklist, which was modified to suit requirements specific to the protocol of cohort studies (see Supplementary material).

### 3.1 Study design

**Figure 1:**
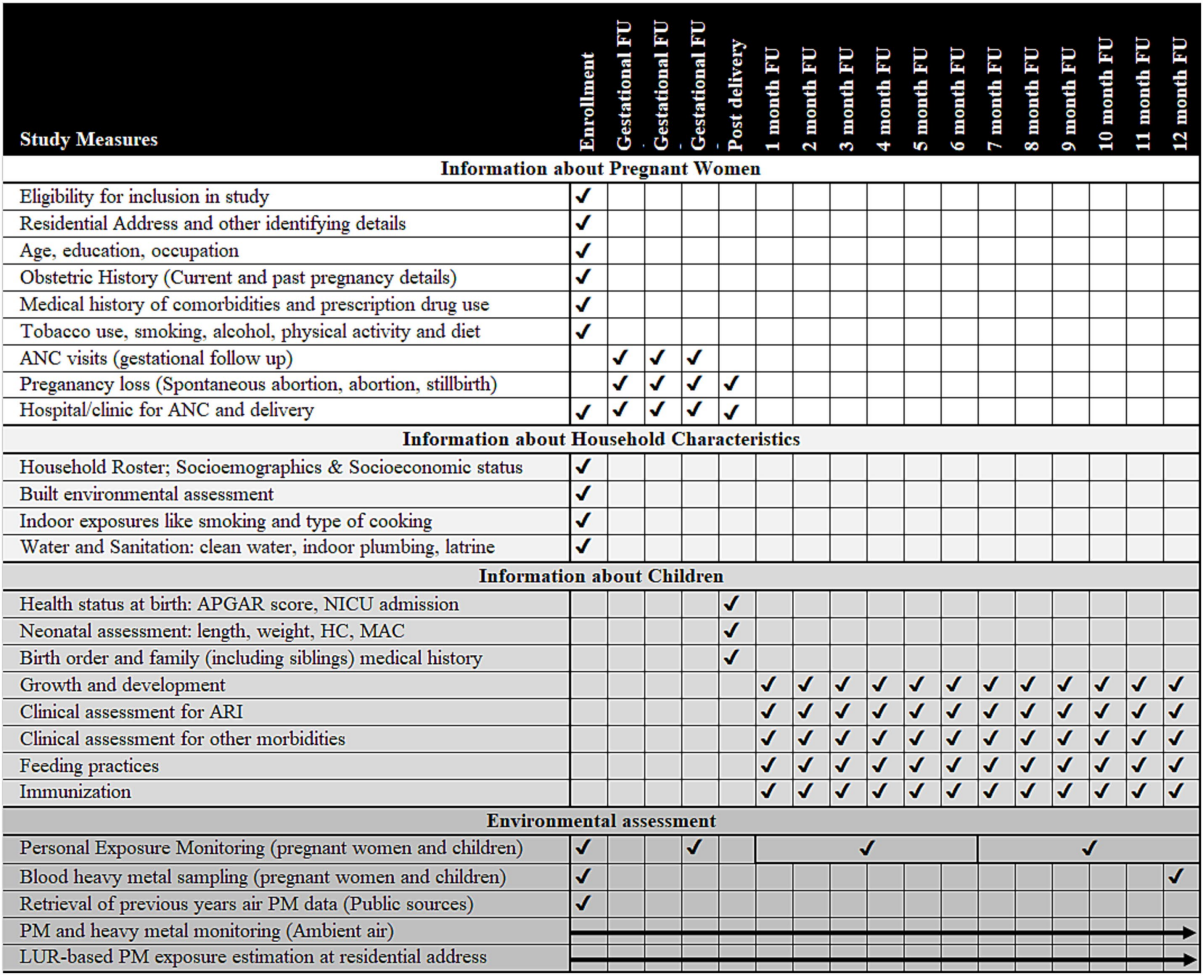
Overview of activities to be conducted in the study (Activities under the study are listed on each row and the timing of each activity is indicated by a tick mark in the cells corresponding to the column headings showing the phase of study such as enrolment, gestational follow-up, post-birth and month-wise follow-up (FU) in the infancy period. Ambient air particulate matter/heavy metal assessment and land-use regression (LUR) modeling will be done over the entire study period and have been shown using arrows. ANC, antenatal care; APGAR, scoring given to child after birth based on appearance, pulse, grimace, activity, and respiration; ARI, acute respiratory infection; HC, head circumference; LUR, land use regression; MAC, mid-arm circumference; NICU, neonatal intensive care unit; PM, particulate matter).

This is the protocol for a population-based prospective cohort study. During the first phase of this study, which will be implemented over three years, we will establish the mother–child birth cohort and conduct regular follow-ups for exposure and clinical assessments of study participants during the antenatal and 1-year postnatal period (see Figure 1). In the next phase, the established cohort will be followed up annually by securing further funding.

### 3.2 Study setting

**Figure 2:**
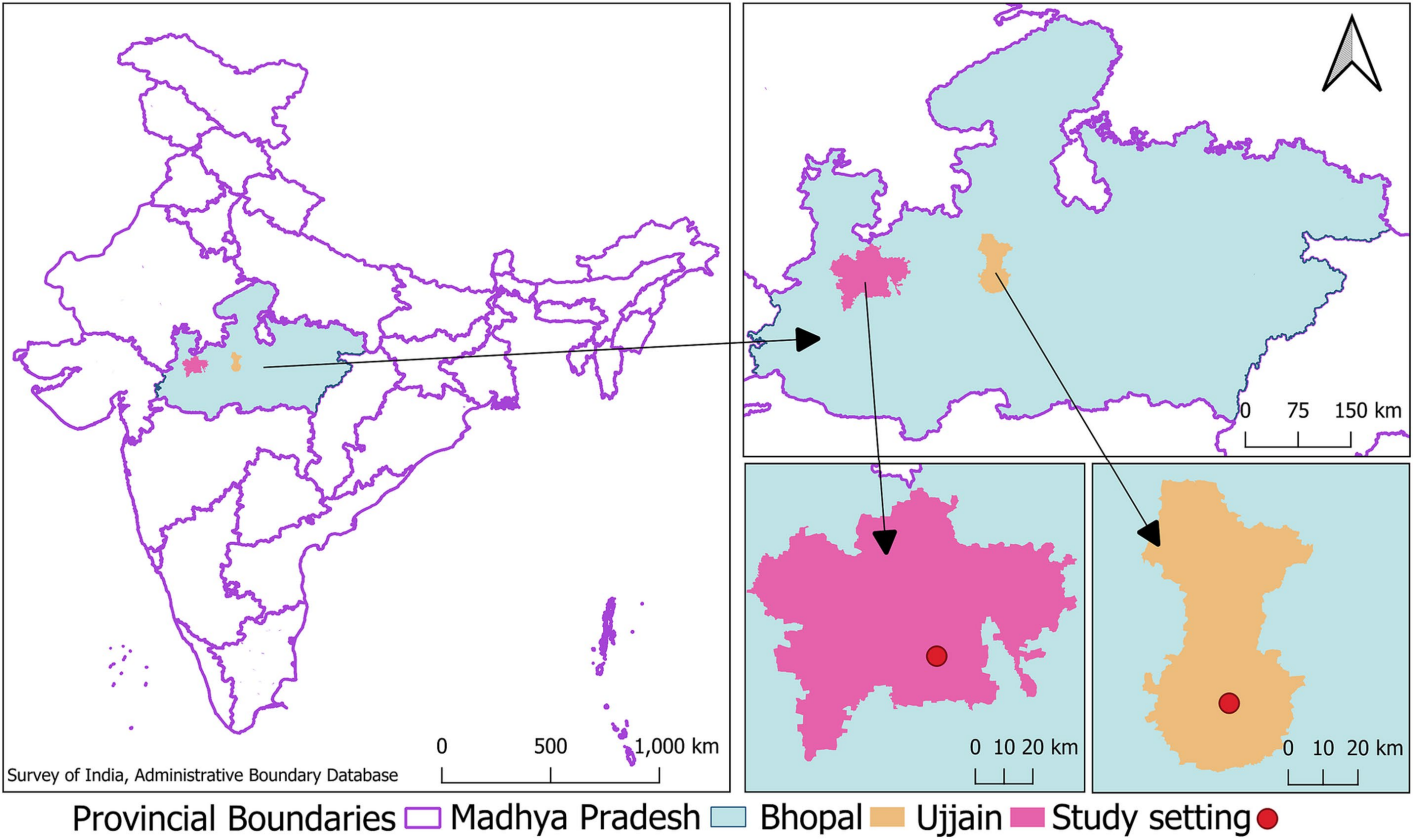
Map showing the location of study cities the image shows clockwise the map of India with the location of the central Indian province of Madhya Pradesh demarcated in green, inset of Madhya Pradesh showing the location of study cities- Bhopal and Ujjain (This image was developed by the research team in the QGIS software (Version 3.28.15). The India map with state and provincial boundaries and point location of Indian District Head Quarters (indicating location of cities) was retrieved from the survey of india- administrative boundary database available at https://onlinemaps.surveyofindia.gov.in/Digital_Product_Show.aspx).

The cohort population will be enrolled within the boundaries of urban local bodies (municipal corporations) of two selected cities (Bhopal and Ujjain) of Madhya Pradesh (MP), a large province located in central India (see Figure 2). The process of finalizing these two cities to establish the cohort has been detailed in Supplementary material (Data Sheet 1). MP is a large province with a total population exceeding 72 million (38), of which around 25% reside in urban areas (39). MP has thirty-two cities, with Bhopal and Ujjain ranking as the second and fifth most populous cities in the province, respectively (40). The child population accounts for 12.02 and 11.45% of the total population of these two cities, respectively (41, 42). The average literacy rate of the total population (Bhopal: 83.47% versus Ujjain: 84.43%, both of which are at a higher level than the Indian average of 74.04%) and proportion of slum population (Bhopal: 26.68% versus Ujjain: 23.32% both of which are at a higher level than the Indian average of 5.41%) in these two cities are comparable, highlighting that the socioeconomic scenario of these two cities are similar (41, 42).

### 3.3 Study population

Consenting pregnant women (*n* = 783 from each city), aged ≥ 18 years, with documented gestational age less than or equal to 16th week, who have been primarily residing in the study cities for at least the past year (and have no imminent plan to shift residence for more than a continuous period of one month away from their current address during the study period) will be enrolled. We will exclude pregnant women who have a history of using any assisted reproductive technology or who have been diagnosed with a high-risk pregnancy where complications are anticipated (43). We will also exclude pregnant women with occupation or lifestyle factors that are reported by previous studies to be sources of exposure to high levels of PM (44–46). Women with pregnancy loss, preterm birth, and term neonates with birth asphyxia and diagnosed congenital anomalies will be identified using relevant records and withdrawn from further follow-up. Details of operational definitions to be used for excluding participants at the time of enrolment and during follow-up phases have been described in Supplementary Table S1.

#### 3.3.1 Sample size calculation

The calculated sample size is 571 mother–child pairs. Our main objective is to investigate the effect of early-life exposure to air pollution on child growth/development. Stunting, a prevalent manifestation of chronic malnutrition, is one of the significant indicators for delay in the achievement of growth/developmental milestones in children (47). Hence, we have calculated the sample size by assuming a power of 80%, a two-sided confidence level of 95%, and by using data available from a recent publication (48). This article estimated an odds ratio (OR) of 1.74 for stunting among under-five children with long-term exposure to PM_2.5_. We applied relevant continuity correction and inflation to account for participant exclusions during follow-up and anticipated non-response/participant attrition. Thus, we will enroll 783 pregnant women in each study city (1,566 participants in total) to establish the final cohort of 571 mother–child pairs. Further details of sample size calculation have been enumerated in Supplementary material (Data Sheet 2). We will continue to use the same cohort for our subsequent objectives.

#### 3.3.2 Sampling strategy

We will obtain information about eligible pregnant women in the study area from community health workers (CHWs), who are usually the first point of contact for antenatal care (ANC) among Indian pregnant women (49, 50). Before the launch of the study, we will identify and enlist, with the help of relevant district-level offices, all the CHWs working in both study cities under the Integrated Child Development Scheme (ICDS) and National Health Mission (NHM) (51, 52). These include Anganwadi workers (AWW) and Accredited Social Health Activists (ASHA), respectively (49, 50). We will approach all CHWs to gain their consent to cooperate, and those willing will be briefed about the study participant enrollment procedure.

We will contact pregnant women identified by CHWs at their homes or at the nearest Anganwadi Centre [community maternal and child care centre in India functioning under the ICDS (53)] to describe our study, confirm their eligibility using a pre-designed checklist, and finally obtain written informed consent. After administration of written informed consent (including consent for follow-up of their child for one year), pregnant women will be enrolled for participation in the study.

### 3.4 Data collection

#### 3.4.1 Exposure data collection

A multi-pronged strategy will be used to collect individual and neighborhood-level exposure data. To begin with, historical data on the neighborhood-level concentration of air PM (PM_10_ and PM_2.5_) covering the two study cities will be retrieved from available open-access sources. For example, in India, in addition to using satellite-derived aerosol optical depth as a source for PM data, the Central Pollution Control Board (CPCB) provides freely available information from its network of fixed-site automatic monitors (continuous ambient air quality monitoring stations - CAAQMS) and gravimetric samplers under the National Air Quality Monitoring Program (NAMP) (54, 55). Using the city-level air PM data, we will develop an exposure database at a high spatial scale using the Land Use Regression (LUR) model for both cities. LUR has been widely used to predict the concentration of air pollutants (measured through distant fixed site monitors and/or remote sensing data) at target locations (i.e., the residential addresses of the selected participants) (56–58). We will develop our LUR model to assign ambient PM_2.5_ and PM_10_ exposure to each participant based on the geocodes of their residence. We will follow the methodologies previously published for urban area LUR models, such as that under the ESCAPE project (59) and for Indian cities (60, 61). Accordingly, in our LUR model, we will use data for a range of geographic, population, and emission source-specific predictor variables retrieved from open access sources, including satellite data, local authorities, and/or collected from field-level verification. For example, data on land use, road network, population density, elevation, meteorological, and other relevant variables will be used, in line with previous studies (56–58). We will adopt a supervised forward addition linear regression approach for model development and perform diagnostics on the fitted models to maintain the linear regression. Finally, we will assess the performance of the developed model by adopting a 10-fold cross-validation approach (62). In addition, we will also measure the personal exposure to air PM (PM_10_ and PM_2.5_) of each enrolled pregnant woman once in each trimester after enrolment to increase the accuracy of PM exposure data while accounting for participant mobility. For these measurements, low-cost battery-operated portable sensors will be used, as has been reported in previous studies (63, 64).

For the heavy metal component of our study, we will conduct biomonitoring of study participants. Blood samples collected from enrolled pregnant women and their live-born children will be analyzed through Inductively coupled plasma optical emission spectroscopy (ICP-OES) using the hot-acid digestion method given by the United States Environmental Protection Agency (USEPA) (65). We will limit our analysis to those heavy metals that are known to adversely affect childhood growth and development and are found in the ambient air of study cities during our air sampling and subsequent composition study. For this, we will refer to previous large-scale studies conducted in Spain, China, and other countries, as well as published systematic reviews (66–70), to compile a list of heavy metals with known adverse effects on childhood growth and development. Simultaneously, we will conduct air PM composition analysis focussing on identifying heavy metals that exceed the permissible limits in the ambient air following CPCB methodology (71) for sampling and, subsequently, USEPA guidelines for laboratory analysis (65).

#### 3.4.2 Outcome and covariates data collection

##### 3.4.2.1 Pregnant women

In the first (baseline) visit to households of eligible and consenting pregnant women, the detailed residential address (including contact details and geographic coordinates), as well as details of the participant and her household members, will be collected at the time of enrolment. Briefly, we will collect information on age, education, occupation, lifestyle factors, medical history (including details of current/past pregnancy and prescription drug use), and relevant family history from the pregnant women. The maternal height and weight will be recorded at the baseline visit. Detailed data about previous pregnancies (if any) will include parity status, birth intervals between consecutive births, and history of anemia/malaria/urinary tract infection/other infections/any other complications in a past pregnancy. In addition, the demographic details, socioeconomic characteristics, water, sanitation, hygiene, and environmental factors related to her household will be collected. The enrolled pregnant women will be followed up once in each trimester in the remaining period of gestation to collect information regarding the occurrence of spontaneous abortion/miscarriage and the details of scheduled antenatal check-ups undergone by the pregnant women. We will note their preference for a hospital/healthcare facility where they would opt for childbirth.

##### 3.4.2.2 Live-born children

###### 3.4.2.2.1 Early neonatal period

According to the information regarding the calculated expected date of delivery (EDD) obtained during the baseline visit, the pregnant women will be contacted within 1 week of the EDD to collect information on pregnancy/birth outcomes (date of childbirth to calculate the child’s chronological age, live birth versus stillbirth, gestational age at birth and congenital anomalies). After getting information about the birth, study staff will visit the hospital/house of the mother to collect anthropometric data of the baby (preferably within 72 h of birth) to supplement the information obtained from hospital/medical records. In cases where the mother/child cannot be contacted within 72 h of birth, we will try to collect relevant data about the child as early as possible before the 1st-month follow-up visit.

###### 3.4.2.2.2 Monthly follow-up of the child till 1st year of age

All eligible live-born children will be followed up at their homes/nearest Anganwadi centre. To ensure timely and smooth data collection, follow-up visits will be scheduled in a manner that these visits coincide with routine house-to-house visits carried out by CHWs under different ongoing national programs [such as Home-based newborn care (HBNC) and Home-Based Care for Young Child Programme (HBYC) under NHM and “Poshan Abhiyan” (72), “Dastak Abhiyan” specific to MP (73)]. In each monthly follow-up visit, anthropometry, achievement of developmental milestones, and acute morbidity profile will be recorded. Additional information about at-birth APGAR score, weight, length, head, and mid-arm circumference will be collected in the 5th visit (child age 1 month). Blood samples for heavy metal analysis and the data on child immunization and feeding practices will be collected in the 17th visit (during the 12th month of the child’s age).

##### 3.4.2.3 Details of data to be collected

The list of domains, variables, and measurement timing of this study are described in subsequent paragraphs and shown in Figure 1.

###### 3.4.2.3.1 Exposure variables

The main exposure/independent variables to be investigated in this study are mean (daily/monthly/annual) concentration of particulate matter (PM_10_ and PM_2.5_) for the specific geo-location of each study participant and mean levels of heavy metal in blood samples of participants (pregnant women and children).

###### 3.4.2.3.2 Study outcomes

The primary study outcomes are related to childhood growth and development, as shown in Tables 1, 2. In addition, we will also estimate the incidence rate of ARIs (number of episodes of ARI during infancy/total number of children under follow-up). We modified the Integrated Management of Childhood Illness definition for ARI [given by WHO/UNICEF (74)] which has been used previously (75) to create an operational definition of ARI for this study. Modification was done by incorporating the signs and symptoms mentioned in the Integrated Management of Neonatal and Childhood Illness (IMNCI), Ministry of Health & Family Welfare, Government of India. Accordingly, ARI will be defined as cough or difficulty in breathing with or without any general danger signs, with or without any chest indrawing, stridor, and fast breathing. Depending upon the site of inflammation determined by the paediatrician based on clinical signs and symptoms, it will be classified as ARI of the upper respiratory tract and ARI of the lower respiratory tract. Further, depending upon the severity, ARI will be classified as per IMNCI guidelines into “No Pneumonia: cough or cold,” “Pneumonia,” and “Severe Pneumonia or Very Severe Disease.” Based on previous research, we will define each episode of ARI to last for two weeks (75). When a child who has had no symptoms for at least one week develops signs and symptoms of ARI, we will treat that as a new episode (75).

**Table 1:** Operational definitions, indicators, and measurement methods to be used for child growth-related outcome measures.

Outcome	Operational definition	Variable/indicator	Measurement method
Small for gestational age (SGA)	As per the WHO definition of SGA given by the 1995 WHO expert committee, infants having a birth weight for gestational age below the 10th percentile based on a sex-specific reference population (139, 140).	Proportion of children diagnosed as SGA at birth (proxy of in-utero growth of the child)	Based on hospital records-verified by study staff
Low birth weight (LBW)	Infants with a birth weight of less than 2,500 g, regardless of gestational age at the time of birth (141, 142).	The proportion of children diagnosed as LBW at birth (proxy of in-utero growth of the child)	Based on hospital records-verified by study staff
Overweight/ obese	Children’s body mass index (BMI - calculated as weight in kilograms divided by height in metres squared) will be plotted on WHO BMI charts and those with a BMI above the 95th percentile will be classified as obese and overweight will indicate those children whose BMI falls between the 85th and 95th percentile (143, 144).	The proportion of overweight/obese (based on BMI for age) infants at the end of the first year of life.	Monthly measurement of height/length, weight, and calculation of BMI
Underweight, wasted and stunted	*Z*-scores for growth indicators (length/height-for-age, weight-for-age, weight-for-length/height, BMI-for-age) will be estimated by comparing each child’s height/length and weight with WHO growth standards as shown in the image below (which has been adapted from the WHO child growth standards: training course on child growth assessment) (145). Based on where their calculated z-scores lie with respect to the median value of the standard reference population, children will be categorized as: Underweight: ‘weight for age’ below minus two standard deviations (-2SD) Severely underweight: ‘weight for age’ below minus three standard deviations (-3SD) Stunted: ‘height/length for age’ below minus two standard deviations (-2SD) Severely Stunted: height/length for age’ below minus three standard deviations (-3SD) Wasted: ‘weight for height/length’ below minus two standard deviations (-2SD)	Proportion of children with abnormal Z-scores for afore-mentioned growth indicators (i.e., underweight, severely underweight, stunted, severely stunted, wasted and severely wasted) at the end of the first year of life.	*Z*-scores for growth indicators (length/height-for-age, weight-for-age, weight-for-length/height, BMI-for-age) obtained from comparison of each child’s height/length and weight with WHO growth standards

**Table 2:** Operational definitions, indicators, and measurement methods to be used for child development-related outcome measures.

Outcome	Operational definition	Variable/Indicator	Measurement method
Development progress	The developmental level of the child will be assigned a score ASQ-3 (146, 147). ASQ-3 has been widely used in many countries as a field-based parent-completed screening tool to assess five developmental domains (148). It has also been previously used and validated in the Indian population (149, 150).	Domain-specific and mean score of ASQ-3 obtained monthly	Ages and stages questionnaires- third edition (ASQ-3) questionnaire (146, 147)
	Developmental Quotient (DQ) is calculated by dividing Developmental Age by Chronological Age and multiplying it by 100 (151, 152). Developmental Age (DA): Each child will be assigned a DA at each follow-up visit for each of the 5 developmental domains, i.e., gross motor, fine motor, adaptive/cognitive, language and personal-social developmental domains. Study staff under the supervision of a paediatrician will estimate DA according to the achievement of domain-specific developmental milestones. Chronological Age (CA): the actual age of the child calculated from his/her date of birth	Proportion of children with delayed achievement of development milestones at the end of the first year of life. Domain-specific monthly developmental level of each infant obtained through Development quotient calculation	Questionnaire developed by authors and pilot-tested in an ongoing built environmental child cohort (84)

###### 3.4.2.3.3 Confounding variables/effect modifiers

We will collect information for a wide range of covariates identified from a detailed literature review. We will collect information using questionnaires and case report forms developed for this study. Wherever possible, relevant questions will be adapted from pre-existing validated questionnaires. To minimize attrition over time, we will ensure that an optimal number of questions will be asked without missing out on important information.

Child-specific details will be retrieved from discussions with care providers and by consulting available hospital records. Briefly, we will record information such as age, gender, birth order, status of immunization [i.e., as per the National Immunization Schedule (NIS) of the Universal Immunization Programme (UIP) in India (76)], history of anaemia and other acute morbidities (defined as per the Integrated Disease Surveillance Project (IDSP) (77) of the Government of India), relevant medical history, including at-birth details of APGAR score, length, weight, head circumference, mid-arm circumference, neonatal intensive care unit (NICU) admission, co-morbidities and family history.

We will collect family-specific information in the form of a household roster detailing the age, education, occupation, relevant medical history, and tobacco/alcohol consumption [using standard questions provided by the WHO STEPwise instrument (78)] of family members. Then, the household socioeconomic status (SES) will be categorized based on scores assigned using the latest revision of the Kuppuswamy Scale (79). Household water sanitation and hygiene assessment will be done using a pre-tested structured questionnaire which the study team has developed by adapting questions from the “Core questions on drinking-water and sanitation for household surveys” given by WHO/UNICEF (80).

In addition to the data collected at baseline, current pregnancy-related information will also be collected from the pregnant women. After birth, during monthly follow-ups with the child, the mother will be asked about breastfeeding and complementary feeding (after the child attains 6 months of age) practices. For data collection on complementary feeding, we will use relevant questions from a questionnaire previously used in Indian settings (81).

Finally, information about other environmental factors, such as the built environment and other indoor exposures, will be collected by adapting questions about built environmental factors such as ventilation, building material, area and construction of the house, and fuels used for cooking or heating from the ‘household questionnaire’ of the National Family Health Survey, India 2019–20 (NFHS – 5) and the ‘house-listing and housing census schedule’ of census 2011 (82, 83). Additionally, we will leverage the field experience gained by the study team in designing and using an exposure questionnaire in a previous cohort study conducted in central India, focussing on the impact of the built environment on population health (84–86).

### 3.5 Data management

We will formulate a Data Collection and Management Committee (DCMC) consisting of the team’s principal investigator and other senior investigators. This committee will ensure that during the implementation of this research, the national guidelines for biomedical research data collection and management (87), are followed stringently to maintain the quality of data collection and analysis. For example, equipment used in the study will be routinely calibrated, and laboratory testing will be conducted using high-quality reagents. Research staff will be adequately trained before the start of project work. We will validate the study questionnaires, which have been developed by adapting questions from standardized tools, and these will be pilot-tested before use. DCMC will also handle requisite communication with the ethics committee and other ethical considerations, including data dissemination. The DCMC will ensure proper management of data. Filled questionnaires will be stored in secure locked cabinets, while online or computer-based information will be kept safe using passwords accessible only to the research team. All data forms will have a unique identifier, which will be used to link collected information on exposures, outcomes, and confounders to ensure accurate data integration while maintaining participant confidentiality. Data entry done by research staff will be supervised by the DCMC, who will randomly verify the entered data to check for data entry mistakes. We will use multiple imputation methods to manage missing data.

### 3.6 Statistical analysis and reporting

The data will be entered using open access online applications from which the data will be downloaded and exported to SPSS statistics software (Version 25) and the latest versions of R packages/R studio for analysis. Data will be summarized using descriptive statistics like mean/standard deviation (SD) or median/Interquartile range. For categorical variables, summary estimates will be reported using frequency with percentages and within two selected study settings. An initial analysis will compare aggregated data between cities with high and low pollution levels. For this, relative risk (RR) and attributable risk (AR) will be calculated through the relevant equations using the probability of outcome (incidence/proportion) among the high pollution group (exposed) divided by the probability of outcome among the low air pollution group (relatively unexposed).

The next level of analysis will be based on the range of the estimates of PM_2.5_ /PM_10_ exposure concentrations and blood heavy metal levels found in the study participants (i.e., pregnant women/live-born children). We will use relevant correlation coefficients to assess possible correlations between environmental variables- PM2.5 /PM10/blood heavy metal levels and the health outcomes – growth indicator related z scores/development quotient/ARI episodes (Pearson or Spearman).

Finally, multivariable logistic regression model analyses will be used to study the relationship between environmental variables- PM_2.5_ /PM_10_/blood heavy metal levels and the health outcomes in binary forms – growth/development/ARI. Briefly, multiple linear mixed-effects regression model analyses will be used to study the relationship between the levels of early-life exposure (concentration of environmental pollutants- PM_2.5_ /PM_10_ concentration/ heavy metals in pregnant women and the child participant- monthly means for the former and cumulative mean- i.e., in-utero maternal exposure summed with infancy period exposure to heavy metal) and child health outcomes- mean anthropometric data (height, weight, head, and mid-arm circumference) and developmental level (obtained from developmental quotient/mean score on ASQ-3 scale) of each participant (live-born children) while adjusting for confounders. Similar regression equations will be used to analyze the association between exposure and delay in achieving growth and developmental milestones. Subsequently, the PM_2.5_, PM_10_ data and other environmental/sociodemographic data will be used in a logistic regression model of ARI symptoms within a distributed lag nonlinear modeling framework (DLNM) (88, 89). This approach will test the associations of PM_2.5_ /PM_10_ exposure over the preceding time periods and the distribution of growth z-scores / occurrences of ARI events (90–92).

Analyses will be performed separately for each child’s morbidity outcomes, such as growth/developmental parameters/domain. All analyses will adjust for different confounding variables included in the study. Details of such confounders for statistical adjustment will consist of gender, family size, socioeconomic status, gynecological/obstetrics/other relevant medical history, and data from environmental assessments of the households (built environmental attributes). We will also adjust for the effect of low birth weight and small-for-gestational-age on the studied association (Supplementary material). We will also conduct sensitivity analyses to test the robustness of our findings, for example, analysing a subset with complete data and comparing the results with the entire dataset where missing data imputations have been done.

### 3.7 Ethical considerations

This study was reviewed and approved by the Institutional Ethics Committee (Human), National Institute for Research in Environmental Health (vide approval letter number: ICMR-NIREH/BPL/IEC/2023–24/1307 dated 16/02/2024). At the beginning of the research, we will obtain written informed consent from the pregnant women for both their and their child’s participation, allowing data collection and scientific dissemination. If we identify any pregnant women or children with signs of illness or health complications during our survey, we will refer them to a nearby government hospital. We will inform the study participants and their families about the study’s final findings through meetings organized in the community with the help of the CHWs. We will also disseminate the findings scientifically through the publication of peer-reviewed articles.

## 4 Discussion

The ELitE birth cohort study will be a relevant addition to the currently sparse evidence base about the long-term effects of early-life exposure to air pollutants among Indian children. Owing to physiological pulmonary adaptation during pregnancy, women inhale a higher volume of air and the pollutants in it in each respiratory cycle (93). Adverse health effects in the mother coupled with the now-acknowledged potential of PM to cross the placenta cause placental malfunctioning as well as other direct and indirect environmental insults to the susceptible developing fetus (94–98). Postnatally, multiple factors such as higher baseline ventilation rates, predominance of mouth-breathing bypassing nasal clearance of particles, and immature immune/pulmonary systems contribute to both higher risk of PM exposure and increased inhaled doses post similar levels of exposure in children who have different deposition and clearance probabilities than adults (99). Hence, pregnant women and children, particularly those living in low-resource, high-exposure settings like India, are especially vulnerable to the adverse health effects of air pollution. Therefore, it is essential to collect prospective longitudinal data to document and understand these impacts over time.

The adverse effects of PM exposure on child growth/developmental trajectory have been shown by epidemiological studies, albeit with contradictory findings (13–17). However, the biological mechanisms mediating these effects are not well explored, leading to perpetuating ambiguity (100). Recently, Sinharoy et al. (101) attempted to elucidate the pathophysiological framework underlying the exposure-response relationship between air pollution and stunting. Authors posited that antenatal exposure via oxidative stress and inflammation leads to mitochondrial dysfunction, decreased DNA methylation, and shortened telomere length, all of which contribute to placental dysfunction and poor foetal growth (101). Further, authors argue that adverse effects on developing immune and pulmonary systems affecting the child’s susceptibility to infections compounded by alterations in appetite versus bodily requirements and dietary metabolite absorptions vis-à-vis loss, particularly that of vitamin D, can contribute to post-natal growth failure leading to stunting (101). Other researchers have also reported similar mechanisms (102, 103). Growth failures due to chronic malnutrition during early childhood can independently translate into developmental delay (104, 105).

On the other end of this spectrum, research has found links between PM exposure and adiposity. The association between PM and obesity is well-established among older children and adults, as shown by a recent review (106). It is said to be mediated via many mechanisms ranging from oxidative stress/inflammation and epigenetic changes to perturbations in metabolic and intestinal flora balance (106). However, the common postulation explaining this dichotomy in the PM effect on growth trajectory reasoned that children with early-life growth restriction are at higher risk of metabolic diseases, including obesity, at a later phase in life as per the developmental origins of health and disease (107, 108). However, recent evidence challenges this mode of reasoning since obesity and higher adiposity have been noted among exposed infants (18, 19). Another line of thinking has evolved, which contemplates that the phenotype of fetal growth restriction in an antenatally exposed mother could be immediately followed by rapid weight gain in the infancy period (109). However, the overall paucity of longitudinal evidence precludes the drawing of any conclusions. The study presented here will help fill in the existing gap in knowledge owing to the scarcity of longitudinal evidence by establishing a birth cohort consisting of mother–child pairs from two urban areas of central India. Although findings are not expected to be generalizable globally, the prospective follow-up data collected in this cohort study will provide strong evidence for planning and policymaking in this field.

This study has multiple advantages owing to its multi-disciplinary research team, which aids in comprehensive information collection and enhances the validity of the study’s findings. Such a strength would be crucial since we deal with child growth and development, a complex multidimensional concept (110, 111). Children are vulnerable to the effects of the complex interplay between a multitude of factors ranging from environmental and psychosocial to genomic insults (112). These factors are hypothesized to begin their effect even before conception (113). Therefore, researchers are increasingly acknowledging the concept of the “exposome,” first introduced in 2005, as the entirety of all non-genomic influences from conception to death owing to the internal effects from the human bodily environment and external influences encompassing harmful exposures to chemical pollutants/infectious pathogens, health behavioral factors linked to tobacco/alcohol consumption, physical activity or diet and even societal, economic, psychological and spiritual factors (114, 115). However, accounting for all potential exposure and confounder variables is a humongous task demanding high-end infrastructure, cost-intensive analytic methods, and high-dimensional data computational facility, all limited in low-resource settings (116). Therefore, although we have not been able to incorporate multiple exposures such as residential greenness, high-decibel community noise, water/food pollutants such as pesticides, endocrine disrupting chemicals, etc., with known effects on child growth/developmental trajectory and morbidity profile (117–123), and “omic” technologies; we have planned for this longitudinal transgenerational study with follow-up at regular intervals and biological sampling to initiate the investigation in this field in India. As evidence from similar cohorts begins to accumulate, the opportunity for robust data analysis will expand. In the future, the evidence base can be strengthened by merging collected exposure and health outcome data with advanced analyses of stored biological samples, similar to the approach used by the EXPOsOMICS research group (124).

A limitation of this exposure-response cohort is the potential of missing personal exposure information in the first trimester. Considering operational feasibility, the 16^th^ week was chosen as the cut-off for pregnant women’s enrolment and the start of exposure assessment in the antenatal period. Previous research has shown that most women in India report their first antenatal care visit within a median time of 16 weeks of gestation, and only 19.6% of pregnancies are detected and registered within the first trimester (125, 126). Hence, it is likely that we will be enrolling most of our pregnant participants in the gestational period of the 12th -16th week, and our field-data-based exposure assessment will fail to account for the complete picture of first-trimester exposure. This can be a significant limitation since it is plausible that the sensitive window for exposure in this context can be during the first trimester. Data analyzed from the INMA Spanish birth cohort showed that the z-score for length at the 6th month of the child’s age reduced by 6% for every 10-μg/m^3^ increase in antenatal NO_2_ exposure during the first trimester (127). Block and Calderón-Garcidueñas (128) also stated that first-trimester exposure to organic constituents of PM, such as polycyclic aromatic hydrocarbons, had a maximal effect on fetal growth when compared with later exposures. This limitation affects birth cohorts in low- and middle-income countries in Asia and Africa. For example, a rural Ghanaian pregnancy cohort investigating air pollution and childhood growth trajectory enrolled pregnant women at or before the 24^th^ week of gestation (15). Authors would have been facing similar constraints as us since the median time to first visit for antenatal care among African women has been reported to be 5 months (129).

We have attempted to overcome this limitation by incorporating past exposure open-access data (such as CPCB ground monitoring stations/satellite-based estimates) of PM_2.5_ into our constructed LUR models. Previous studies have used this method. For example, the recently published Californian population-based prospective pregnancy cohort study (MADRES) enrolled pregnant women with a gestational age of less than 30 weeks and obtained air pollution exposure information using data from the United States Environmental Protection Agency Air Quality System to model daily participant exposure beginning from 2 years before pregnancy (130). However, using only LUR modeling-based exposure assessment suffers from ecological fallacy (131). Final estimation is highly dependent upon the density of monitoring stations from which PM data can be used to train the models and the granularity of data for predictor variables and, thus, cannot accurately differentiate between individual-specific exposure, particularly when their residential addresses are clustered close together (132, 133). Hence, data triangulation by adding personally measured and biomonitoring data would increase confidence in the overall exposure assessment (134). We have thus adopted a multi-pronged strategy to estimate the study participants’ accurate exposure information.

Finally, to ensure the highest level of internal validity of our study, we will attempt to overcome the biases inherent in adopting a cohort study design (135, 136). For example, to control for attrition bias, loss-to-follow-up will be minimized through better rapport building with the study participants with the support of CHWs. Published evidence acknowledges the crucial role played by CHWs, who are usually residents of the locality where they work and, hence, have an established rapport with their beneficiaries (137, 138). By seeking the active cooperation of CHWs through appropriate incentivization, we will be leveraging this trust built with the community. Hence, we expect attrition to be minimal in the study. In addition, we will also systematically document the characteristics (including the reasons for withdrawal) of those participants who decline further follow-up. Although not wholly amenable to control, other selection biases like non-response bias and healthy entrant bias will be accounted for by asking for and recording relevant information about those pregnant women who refuse to participate in the study when approached for consent (135, 136). Similarly, information and confounding bias will be minimized by ensuring the use of validated pilot-tested questionnaires and appropriate collection of data on exposure, outcome, and confounding variables by trained research staff (135, 136).

## 5 Conclusion

This manuscript outlines the methodology for establishing an Indian mother–child air pollution birth cohort and its follow-up. The study addresses critical gaps in existing evidence on the adverse effects of early-life exposure to air PM and its heavy metal constituents among Indian children. While resource limitations may restrict comprehensive exposure assessment and reduce the generalisability of our findings to all sub-populations within India and other low- and middle-income countries, the study’s strengths will provide a valuable epidemiological foundation for advancing knowledge in this area. By detailing our rigorously planned study methods and outcome measures, which align with established and ongoing birth cohorts, we aim to serve as a foundation for future cohorts. Collectively, such efforts can generate robust evidence to inform context-specific policies and the development of effective prevention and mitigation strategies.

## Glossary

ANC: Antenatal Care
APGAR: Scoring Given to Child after Birth based on Appearance, Pulse, Grimace, Activity, and Respiration
AR: Attributable Risk
ARI: Acute Respiratory Infection
ASHA: Accredited Social Health Activists
ASQ-3: Ages and Stages Questionnaire- Third Edition
AWW: Anganwadi Workers
AQI: Air Quality Index
BMI: Body Mass Index
CAAQMS: Continuous Ambient Air Quality Monitoring Stations
CA: Chronological Age
CHW: Community Health Workers
CPCB: Central Pollution Control Board
DA: Developmental Age
DCMC: Data Collection and Management Committee
DNA: Deoxyribonucleic Acid
DQ: Developmental Quotient
DLNM: Distributed Lag Non-Linear Model
EDD: Expected Date of Delivery
EDXRF: Energy-Dispersive X-Ray Fluorescence
GIS: Geographic Information System
HBNC: Home-Based Newborn Care
HBYC: Home-Based Care for Young Child Programme
HVAS: High Volume Air Sampler
ICD: International Classification of Disease
ICDS: Integrated Child Development Scheme
ICP-OES: Inductively Coupled Plasma Optical Emission Spectroscopy
IDSP: Integrated Disease Surveillance Project
IMNCI: Integrated Management of Neonatal and Childhood Illness
LBW: Low Birth Weight
LMP: Last Menstrual Period
LUR: Land Use Regression
MP: Madhya Pradesh
NAAQS: National Ambient Air Quality Standards
NFHS: National Family Health Survey
NHM: National Health Mission
NICU: Neonatal Intensive Care Unit
NIS: National Immunization Schedule
NO_2_: Nitrogen Dioxide
OR: Odds Ratio
PM: Particulate Matter
PM_10_: Particulate Matter with Aerodynamic Diameter < 10 μm
PM_2.5_: Particulate Matter with Aerodynamic Diameter < 2.5 μm
RR: Relative Risk
SD: Standard Deviation
SDG: Sustainable Development Goal
SES: Socio-Economic Status
SGA: Short For Gestational Age
SO_2_: Sulphur Dioxide
UIP: Universal Immunization Programme
UNICEF: United Nations Children’s Fund
UNEP: United Nations Environment Programme
USEPA: United States Environmental Protection Agency
WHO: World Health Organisation
Measurement units
m: Metre
ml: Millilitre (1 mL = 10^−3^litre)
μm: Micrometre (1 μm = 10^−6^ m)
kg: Kilogram
g: Gram (1 g = 10^−3^kilogram)
μg: Microgram (1 μg = 10^−6^ g).
