# Supplementary material for: A protocol for estimating health burden posed by early life exposure to ambient fine particulate matter and its heavy metal composition: a mother–child birth (ELitE) cohort from Central India
Source: Front Public Health. 2025 May 20;13:1485417. doi: 10.3389/fpubh.2025.1485417 (PMC12130030; doi:10.3389/fpubh.2025.1485417)
Supplement: Supplementary file 1 [file Data_Sheet_1.docx]

**Additional File 1: Selection of cities for establishment of cohort**

We used open-access ambient air quality data provided by the Central Pollution Control Board (CPCB), the apex organisation for environmental monitoring in India, to select our study locations. CPCB uses a complex algorithm to calculate the AQI values and classify the ambient air quality into six categories ranging from “severe” (with AQI in the range of 401-500) to “good” (with AQI in the range of 0-50). The data of the AQI levels of Indian cities where CPCB has installed fixed-site monitors is available online. Similarly, CPCB also publishes yearly annual average concentration of particulate matter measured under the National Air Quality Monitoring Programme (NAMP) for all stationary monitors located in various districts/cities of India. We retrieved these open-access data to systematically select our study locations.

At the outset, we chose to establish our cohort in the central Indian province of Madhya Pradesh (MP). This decision was taken because limited evidence is reported from this region of the country in published literature. Further, our institution is located in MP which will facilitate the establishment of the cohort and help in minimizing attrition. Thus, we created a list of cities of MP using the latest available census data. We then filtered out those cities that had a population of less than 5 lakhs as per the 2011 census since it would be difficult to enroll the required sample size of pregnant women. We, then, downloaded and tabulated the average AQI values of each remaining city of MP from 2019 to 2023. In addition, we also used the latest available NAMP annual average concentration of particulate matter in these cities. Finally, we retrieved the number of real-time sensor-based monitors / gravimetric samplers installed in each city and used to estimate each city's average air quality.

Using these three parameters, we classified the cities in MP into two categories representing “high” and “low” air pollution levels. From the “high” group, we randomly selected one city (Bhopal). From the “low” group, we purposively selected Ujjain as it has a similar sociodemographic and cultural background. Thus, we selected two cities - Bhopal and Ujjain from the central Indian province of MP to establish the ELitE cohort. Although we will be measuring exposure and outcome for each participant and analysing these values as a continuous quantitative variable, selecting participants from two cities with documented high and low AQI levels will enable us to enrol participants with maximal variation in air particulate matter (PM) exposure.
