# Supplementary material for: A protocol for estimating health burden posed by early life exposure to ambient fine particulate matter and its heavy metal composition: a mother–child birth (ELitE) cohort from Central India
Source: Front Public Health. 2025 May 20;13:1485417. doi: 10.3389/fpubh.2025.1485417 (PMC12130030; doi:10.3389/fpubh.2025.1485417)
Supplement: Supplementary file 2 [file Data_Sheet_2.docx]

Supplementary Material

# Sample Size Calculation

For sample size calculation we used the formulae given by Fleiss (2003) (1) for detecting a difference between two proportions as shown below:

$$n^{'}=\left[ \frac{\left( Z_{\alpha/2}\sqrt{2\bar{P}\left( 1-\bar{P} \right)}+Z_{\beta}\sqrt{P_{1}\left( 1-P_{1} \right)+P_{2}\left( 1-P_{2} \right)} \right)^{2}}{\left( P_{2}-P_{1} \right)^{2}} \right]\& CC=\left[ \frac{\left( 1+\sqrt{1+\frac{4}{n^{'}\left( P_{2}-P_{1} \right)}} \right)^{2}}{4} \right]$$

Where $n^{'}$ represents the sample size calculated without continuity correction (CC); z alpha for 5% level of significance (*Z_α/2_*) is 1.96; z beta for 80% power (*Z_β_*) is 0.84; Proportion of event in the unexposed group (*P_1_*) is 0.175^[[1]](#footnote-1)^; Proportion of event in the exposed group (*P_2_*) is calculated using an Odds ratio (*OR*) of 1.74^[[2]](#footnote-2)^ using the formula $P_{2} = (P_{1}*OR) /(P_{1}*OR+ (1- P_{1}))$; and $\overline{P}$ is the average of P_1_ and P_2_.

Then we applied continuity correction to minimise the difference between the proportion values used based on a sample versus the real proportion of events in the population using the following formula (1):

$$n= \left( n^{'}\times\left[ CC \right]\times\left[ 1+\frac{Extra}{100} \right]X DEFF \right)$$

Where *n* is the final sample size calculated by applying continuity correction (CC); *Extra* represents a total of 37.15% inflation to account for non-response /Attrition rate (20%), for exclusion of spontaneous abortions & stillbirths (8.3%), pre-term birth (7%) & congenital anomalies (1.85%)^[[3]](#footnote-3)^; and Design effect (*DEFF*) of 1.77^[[4]](#footnote-4)^

References:

1. Fleiss JL. “Statistical Methods for Rates and Proportions, Third Edition.,” In: Fleiss JL, Levin B, Paik M, editors. (2003). p. 187–233 doi: 10.1002/0471445428.ch9

2. Li X, Li Y, Yu B, Nima Q, Meng H, Shen M, Zhou Z, Liu S, Tian Y, Xing X, et al. Urban-rural differences in the association between long-term exposure to ambient particulate matter (PM) and malnutrition status among children under five years old: A cross-sectional study in China. *J Glob Health* 13:04112. doi: 10.7189/jogh.13.04112

3. Kuppusamy P, Prusty RK, Chaaithanya IK, Gajbhiye RK, Sachdeva G. Pregnancy outcomes among Indian women: increased prevalence of miscarriage and stillbirth during 2015–2021. *BMC Pregnancy Childbirth* (2023) 23:150. doi: 10.1186/s12884-023-05470-3

4. Kannaujiya AK, Kumar K, Upadhyay AK, McDougal L, Raj A, James KS, Singh A. Effect of preterm birth on early neonatal, late neonatal, and postneonatal mortality in India. *PLOS Glob Public Health* (2022) 2:e0000205. doi: 10.1371/journal.pgph.0000205

5. Bhide P, Kar A. A national estimate of the birth prevalence of congenital anomalies in India: systematic review and meta-analysis. *BMC Pediatr* (2018) 18:175. doi: 10.1186/s12887-018-1149-0

6. Hulland EN, Blanton CJ, Leidman EZ, Bilukha OO. Parameters associated with design effect of child anthropometry indicators in small-scale field surveys. *Emerg Themes Epidemiol* (2016) 13:13. doi: 10.1186/s12982-016-0054-y

1. We assumed that the proportion of events in the unexposed group [i.e., control population exposed to low air pollutant concentrations] was 17.5% based on the prevalence of stunting in the control/low-exposure group reported by Li et al.,2023 (2). [↑](#footnote-ref-1)
2. Then, we used the odds ratio of 1.74 reported by the same study by Li et al.,2023 (2). for the risk of stunting among children under five years old per 10 microgrammes per cubic metre (μg/m^3^) increase in long-term exposure to ambient air pollution PM_2.5_. Accordingly, the proportion of events in the exposed group was calculated to be 24.7%. [↑](#footnote-ref-2)
3. Since our primary objective is to follow live births until 1 year of age for outcome assessment, we inflated the sample size to account for 8.3%incidence of pregnancy loss (spontaneous abortions, miscarriage, and stillbirth in clinically recognized Indian pregnancies as per the findings from the fifth (2019-21) round of National Family Health Survey (NFHS-5) (3), and an additional 20% to account for non-response / attrition. In addition, we plan to exclude children born pre-term and those with congenital anomalies (since these factors can confound the association between air pollution and child growth/development). Thus, we will also inflate the sample size to be able to exclude pre-term births (7% prevalence calculated by Kannaujiya et al., 2022 using NFHS data) (4) and congenital anomalies (1.85% pooled prevalence reported by the meta-analysis of Bhide and Kar 2018) (5). [↑](#footnote-ref-3)
4. Design effect for this study has been assumed to be 1.77 based on the median design effect for childhood stunting-related field data collection reported by Hulland et al., 2016 based on a literature review and modelling of 380 studies from 28 countries (6). [↑](#footnote-ref-4)
