# Supplementary material for: A protocol for estimating health burden posed by early life exposure to ambient fine particulate matter and its heavy metal composition: a mother–child birth (ELitE) cohort from Central India
Source: Front Public Health. 2025 May 20;13:1485417. doi: 10.3389/fpubh.2025.1485417 (PMC12130030; doi:10.3389/fpubh.2025.1485417)
Supplement: Supplementary file 3 [file Table_1.docx]

Supplementary Material- Additional File 3

# STROBE Checklist (Modified to suit requirements specific to the *protocol* of cohort studies)

|  | Item No | Recommendation | Page number of protocol |
| --- | --- | --- | --- |
| **Title and abstract** | 1 | (*a*) Indicate the study’s design with a commonly used term in the title or the abstract | Page number: 1 |
|  |  | (*b*) Provide in the abstract an informative and balanced summary of what was done and what was found | Page number: 1 - 2 |
| Introduction | | |  |
| Background/rationale | 2 | Explain the scientific background and rationale for the investigation being reported | Page number: 2 |
| Objectives | 3 | State specific objectives, including any prespecified hypotheses | Page number: 2 |
| Methods | | |  |
| Study design | 4 | Present key elements of study design early in the paper | Page number: 3 |
| Setting | 5 | Describe the setting, locations, and relevant dates, including periods of recruitment, exposure, follow-up, and data collection | Page number: 4 |
| Participants | 6 | (*a*) Give the eligibility criteria and the sources and methods of selection of participants. Describe methods of follow-up | Page number: 4 |
|  |  | (*b*) For matched studies, give matching criteria and the number of exposed and unexposed | Not Applicable |
| Variables | 7 | Clearly define all outcomes, exposures, predictors, potential confounders, and effect modifiers. Give diagnostic criteria, if applicable | Page number: 6 |
| Data sources/ measurement | 8* | For each variable of interest, give sources of data and details of methods of assessment (measurement). Describe comparability of assessment methods if there is more than one group | Page number: 6 – 8 |
| Bias | 9 | Describe any efforts to address potential sources of bias | Page number: 10 |
| Study size | 10 | Explain how the study size was arrived at | Page number: 4 |
| Quantitative variables | 11 | Explain how quantitative variables were handled in the analyses. If applicable, describe which groupings were chosen and why | Page number: 6 – 8 |
| Statistical methods | 12 | (*a*) Describe all statistical methods, including those used to control for confounding | Page number: 7 – 8 |
|  |  | (*b*) Describe any methods used to examine subgroups and interactions | Page number: 7 – 8 |
|  |  | (*c*) Explain how missing data were addressed | Page number: 7 |
|  |  | (*d*) If applicable, explain how loss to follow-up was addressed | Page number: 5 – 6 |
|  |  | (*e*) Describe any sensitivity analyses | Page number: 7 |
| Results- | | | Not Applicable since this manuscript describes only the protocol of a cohort |
| Discussion | | |  |
| Key results | 18 | Summarise key results with reference to study objectives | Page number: 8 – 10 |
| Limitations | 19 | Discuss the limitations of the study, taking into account sources of potential bias or imprecision. Discuss both the direction and magnitude of any potential bias | Page number: 9 – 10 |
| Interpretation | 20 | Give a cautious overall interpretation of results considering objectives, limitations, multiplicity of analyses, results from similar studies, and other relevant evidence | Page number: 10 |
| Generalisability | 21 | Discuss the generalisability (external validity) of the study results | Page number: 10 |
| Other information | | |  |
| Funding | 22 | Give the source of funding and the role of the funders for the present study and, if applicable, for the original study on which the present article is based | Page number: 10 |

*Give information separately for exposed and unexposed groups.
